# Anti-Mayaro virus activity of *Cassia australis* extracts (Fabaceae, Leguminosae)

**DOI:** 10.1186/s13071-014-0537-z

**Published:** 2014-11-27

**Authors:** Kassia C W Spindola, Naomi K Simas, Tiago S Salles, Marcelo D F de Meneses, Alice Sato, Davis Ferreira, Wanderson Romão, Ricardo M Kuster

**Affiliations:** Natural Product Research Institute, Center of Health Sciences, Federal University of Rio de Janeiro, Rio de Janeiro, Brazil; Natural Product and Food Department. Center of Health Sciences, Federal University of Rio de Janeiro, Rio de Janeiro, Brazil; Microbiology Institute, Virology Department, Federal University of Rio de Janeiro, Rio de Janeiro, Brazil; Chemistry Institute, Biochemistry Department, Federal University of Rio de Janeiro, Rio de Janeiro, Brazil; Botanical Department. Center of Biological and Health Sciences, Federal University of the State of Rio de Janeiro, Rio de Janeiro, Brazil; Petroleomic and Forensic Laboratory, Department of Chemistry, Federal University of Espírito Santo, 29075-910 Vitória, ES Brazil

**Keywords:** *Cassia australis*, Flavonoids, Tannins, Antiviral, MAYV, Larvicidal activity, *Aedes aegypti*

## Abstract

**Background:**

The arthropod-borne Mayaro virus (MAYV) causes ‘Mayaro fever’, a disease of medical significance, primarily affecting individuals in permanent contact with forested areas in tropical South America. Studies showed that the virus could also be transmitted by the mosquito *Aedes aegypti*. Recently, MAYV has attracted attention due to its likely urbanization. To date, there are no drugs that can treat this illness.

**Methods:**

Fractions and compounds were obtained by chromatography from leaf extracts of *C. australis* and chemically identified as flavonoids and condensed tannins using spectroscopic and spectrometric techniques (UV, NMR, and ESI-FT-ICR MS). Cytotoxicity of EtOAc, *n-*BuOH and EtOAc-Pp fractions were measured by the dye-uptake assay while their antiviral activity was evaluated by a virus yield inhibition assay. Larvicidal activity was measured by the procedures recommended by the WHO expert committee for determining acute toxicity.

**Results:**

The following group of substances was identified from EtOAc, *n-*BuOH and EtOAc-Pp fractions: flavones, flavonols, and their glycosides and condensed tannins. EtOAc and *n-*BuOH fractions inhibited MAYV production, respectively, by more than 70% and 85% at 25 μg/mL. EtOAc-Pp fraction inhibited MAYV production by more than 90% at 10 μg/mL, displaying a stronger antiviral effect than the licensed antiviral ribavirin. This fraction had an excellent antiviral effect (IC_90_ = 4.7 ± 0.3 μg/mL), while EtOAc and *n-*BuOH fractions were less active (IC_90_ = 89.1 ± 4.4 μg/mL and IC_90_ = 40.9 ± 5.7 μg/mL, respectively).

**Conclusions:**

*C. australis* can be used as a source of compounds with anti-Mayaro virus activity. This is the first report on the biological activity of *C. australis*.

## Background

In Brazil, MAYV is endemic in the Amazon region, but Mayaro fever outbreaks have occurred in other regions in Brazil [1,2]. Most arboviruses isolated in the Amazon region are maintained in nature by different life cycles, involving different vectors and vertebrate hosts. Oropouche virus, for example, is transmitted to humans in urban areas by the midges *Culicoides paraensis*, and vertebrates such as sloths, monkeys and birds play a role in the maintenance of the virus cycle [3]. Likewise, Mayaro and yellow fever viruses are transmitted by the mosquito *Haemagogus janthinomys* in the jungle, and monkeys are the main hosts [4]. On the other hand, dengue virus has a simpler cycle whereby the serotypes are directly transmitted to humans by *Aedes aegypti* mosquito bites [5]. In addition, imported MAYV cases in other countries from tourists who visited the Amazon region have been described [6]. MAYV can also be transmitted by the vectors *Aedes aegypti* and *A. albopictus*, which raises the concern for urban areas. It is very important to point out that MAYV is closely related to the Chikungunya virus, also transmitted by the mosquitoes *Aedes aegypti* and *A. albopictus*, which has recently reached Europe and the Americas and is now counting nearly 800 of autochthones infections in Brazil since the first detected case in this country in August 2014 [7,8].

Natural products are becoming very attractive because of their low cytotoxicity, the rapid degradation in the environment, and because of the complexity of the chemistry in these products, that should limit resistance and increase the applicability of use, such as vector control studies [9].

The majority of the available antiviral drugs are concentrated in a small number of viruses, such as HIV, Herpes and Influenza [10]. Nevertheless, research efforts to explore the potential of natural products as sources of novel low toxicity and high selectivity antiviral substances have increased lately [11]. Because there are many approaches for the use of natural products, the modes of action or the active components they contain and the metabolic pathways they interact must be studied. This can be accomplished initially by *in vitro* studies such as the cell culture approach in this paper.

The genus *Cassia* (Fabaceae, Leguminosae) comprises more than 600 species including shrubs, trees and herbs, distributed in tropical and subtropical regions all over the world. The separation of the genus *Senna* from the genus *Cassia* has been, and still is, the subject of many discussions [12]. The species under study was originally classified as *Cassia australis* Vellozo and in 1982 by Irwin and Barneby it was transferred to *Senna* with the name: *Senna australis* (Vell.) H.S.Irwin & Barneby. Afterwards, this species has been renamed *Senna appendiculata* (Vogel) [13].

In Brazilian ecosystems, particularly in the Atlantic forest, the genus *Senna* is widespread, with some species in the Southeast greatly appreciated for the beauty of its flowers,and therefore widely used as ornamental plants [14]. Due to the traditional use, several species, many already described in the literature, are medicinally used worldwide [15–20]. *Cassia australis* is a medium sized shrub and may reach up to 2.5 meters of diameter. It occurs at Brazilian coast sandbank, mainly in Rio de Janeiro, Espirito Santo, Bahia, Sergipe, Alagoas and Pernambuco states [21]. The genus *Cassia* is known for the presence of a variety of compounds. Anthraquinones are the main class of compounds isolated from it [17,22–25]. However, previous investigations led to the isolation of flavonoids [26–29], piperidine alkaloids [30], stilbenoids [30] and aliphatic esters [16]. So far, there is no paper about the phytochemistry and biological activity of *Cassia australis*.

For most mosquito-transmitted viruses, there are no licensed antiviral drugs or vaccines available. MAYV is an example of an arthropod-borne virus, mainly found in South America tropical areas, which affects primarily individuals in permanent contact with forested areas and causes the Mayaro fever. In this study, EtOAc, *n-*BuOH and EtOAc-Pp fractions containing flavonoids and other classes of phenolics compounds were obtained from the leaves of *Cassia australis* Vellozo and investigated for their *in vitro* antiviral activity against MAYV replication in Vero cells and larvicidal activity against *Aedes aegypti* larvae.

## Methods

### Plants, cells and viruses

*Cassia australis* leaves were collected in December 2008 in Rio de Janeiro State, and identified by Alice Sato. Voucher specimens (No. 652HUNI) are deposited in the herbarium of the University of Rio de Janeiro (UNIRIO), Brazil.

Vero cells (African green monkey kidney, ATCC CCL-81) were maintained at 37°C, 5% CO_2_, in Dulbecco’s modified Eagle’s medium (DMEM) (Life Technologies, USA) supplemented with 5% fetal bovine serum (Cultilab, BRA), 50 IU/mL of penicillin, and 50 μg/mL of streptomycin (Sigma-Aldrich, USA). Mayaro virus (ATCC VR-66, lineage TR 4675) was propagated in Vero cells and viral stocks kept at −70°C until use.

*Aedes aegypti* eggs were obtained at the Brazilian Army Institute of Biology. They were kept in the tray containing tap water at optimal conditions (28 ± 1°C). After 48 hrs of incubation, the eggs were used. The 4th instar larvae were used in the study.

### Extraction, fractionation, and purification for achievement of fractions and compounds

Air-dried leaves (850 g) were extracted with MeOH:H_2_O (8:2) at room temperature by static maceration over 10 days. After concentration under reduced pressure, the methanol extract (25 g) was suspended in MeOH:H_2_O (9:1), and partitioned with hexane. After removal of the methanol from the defatted extract, the remaining aqueous solution was partitioned successively with CH_2_Cl_2_, EtOAc, and *n-*BuOH. Two grams of the EtOAc extract, soluble in H_2_O:MeOH (9:1), were applied to a XAD-2 column (procedure A), and chromatographed in a stepwise gradient with H_2_O:MeOH (10:0/0:10). 150 ml of each combination of solvents were eluted through the column and fractions of 150 ml were collected. The fraction obtained with 100% of water was named EtOAc-Pp. From H_2_O:MeOH (50:50) fraction, after chromatography on Sephadex LH-20 (MeOH:H_2_O – 1:1, mobile phase) – (procedure B), the flavone tricetin-4′-methoxy-3′-β-glucoside [31] was obtained. The same procedures A and B were applied to *n-*BuOH extract to obtain the flavone vicenin-2 [32].

### Reverse-phase HPLC-DAD-UV, TLC, NMR and ESI-(−)-FT-ICR MS analyses

HPLC-DAD-UV (High Performance Liquid Chromatography with Diode Array Detector), TLC (Thin Layer Chromatography), NMR (Nuclear Magnetic Resonance) and ESI(−)-FT-ICR MS (Electrospray ionization with Fourier Transform Ion Cyclotron Resonance Mass Spectrometry) were used to analyze the chemical composition of EtOAc, *n-*BuOH, EtOAc-Pp fractions and compounds isolated from them.

The mobile phase for HPLC-DAD analysis consisted of solvent (A) 1% phosphoric acid in water and solvent (B) 1% phosphoric acid in methanol and was used under the following gradient: 5% of B to 70% of B in A for 55 min. The flow rate was 1 mL/min and the injection volume 20 μL. The UV–vis spectra were recorded from 210 to 400 nm and the detector focused on 254 and 365 nm. TLC was performed on silica gel plates 60 F_254_ (Merck, 20×20 cm, 0.5 mm thickness), using *n-*butanol-water-acetic acid (4:5:1) and chloroform-methanol (9:1) as mobile phases. After elution, TLC plates were observed under 254 nm UV light and then sprayed successively with solutions of NP (2-aminoethyldiphenylborinate 1% in methanol) and PEG-4000 (polyethylene glycol 5% in ethanol) (both by Sigma-Aldrich, USA) before examination under 365 nm UV light. *Cassia australis* extracts and pure compounds were analyzed by an ultra-high resolution and accuracy mass spectrometer (model 9.4 T Solarix, Bruker Daltonics, Bremen, Germany). Briefly, the samples were dissolved in acetonitrile/ammonium hydroxide (99.9/0.1 v/v %) mixture to a final concentration of 10 μg mL^−1^. The mass spectrometer was set to operate in negative ion mode, ESI(−), over a mass range of m/z 200–2000. The parameters of the ESI(−) source were as follows: nebulizer gas pressure of 0.5-1.0 bar, capillary voltage of 3–3.5 kV, and transfer capillary temperature of 250°C. The spectrum was processed using the Compass Data Analysis software package (Bruker Daltonics, Bremen, Germany). A resolving power, m/Δm_50%_ ≈ 500 000, in which Δm_50%_ is the full peak width at half-maximum peak height, of m/z ≈ 400 and a mass accuracy of <1 ppm provided the unambiguous molecular formula assignments for singly charged molecular ions. Elemental compositions of the compounds were determined by measuring the m/z values. NMR analysis (^1^H-NMR, COSY, HSQC and HMBC) were recorded on Varian spectrometer MR-400 operating at 400 MHz. The samples were solubilized in DMSO-d_6_ and TMS was used as external standard.

Final compound analysis was performed by NMR (DMSO-d_6_), FT-ICR-ESI-MS, UV spectral analysis, and by comparison with literature values.

### Cytotoxicity assay

Cytotoxicity analysis was performed using the dye-uptake method modified from Borenfreund and Puerner [33]. Vero cells grown in 96-well microplates were treated with culture media containing different concentrations of the substances. After 24 hours, the medium was replaced by a solution of 50 μg/mL neutral red, the cells were incubated for 3 hours at 37°C, 5% CO_2_ and then fixed and extracted with 20% formaldehyde and 50% methanol, 1% acetic acid. Absorbance was read at 490 nm, using a spectrophotometer, to detect neutral red incorporation by living cells. Absorbance results were used to calculate, by regression analysis, the concentrations of the tested substances capable of reducing cell viability by 50% and 90% relative to controls (CC_50_ and CC_90_, respectively).

### Antiviral activity assay

For the antiviral activity, confluent Vero cell monolayers grown in 24-well plates were infected with MAYV (multiplicity of infection = 0.1) for 1 hour, then rinsed with PBS and treated for 24 hours (at 37°C and 5% CO_2_) with different concentrations (0–100 μg/ml) of the substances diluted in culture medium. After treatment, culture supernatants were recovered and used for titration of extracellular infectious virus particles. Ribavirin (Sigma-Aldrich, USA) was used as positive control for MAYV replication inhibition. For each substance or extract, IC_50_ and IC_90_ values were calculated and used to obtain a selectivity index (SI), expressed as the ratio CC_50_/IC_50_, and to estimate relative potency (RP) as the ratio between ribavirin (reference substance) IC_90_ and the tested substance’s IC_90_. Results were presented as mean inhibitory/cytotoxic concentration ± SD, and *t*-tests were used to evaluate the statistical significance of treatments relative to controls. P-values <0.05 were considered statistically significant.

### Virus yield assay

For virus titration, confluent cell monolayers in 24-well plates were infected with serial dilutions of recovered supernatants from the assays for 1 hour at 37°C, 5% CO_2_. After inoculum removal, cells were rinsed with PBS and the monolayer was incubated with fresh medium with 2% carboxymethylcellulose (Sigma-Aldrich, USA) for 48 hours at 37°C, 5% CO_2_. Finally, cells were fixed with 20% formaldehyde and stained with 0.5% crystal violet in 20% ethanol, and viral plaques were counted.

## Results

### Flavonoid aglycones, flavonoid glycosides and tannins were found in extracts of *C. australis* leaves

**Figure 1:**
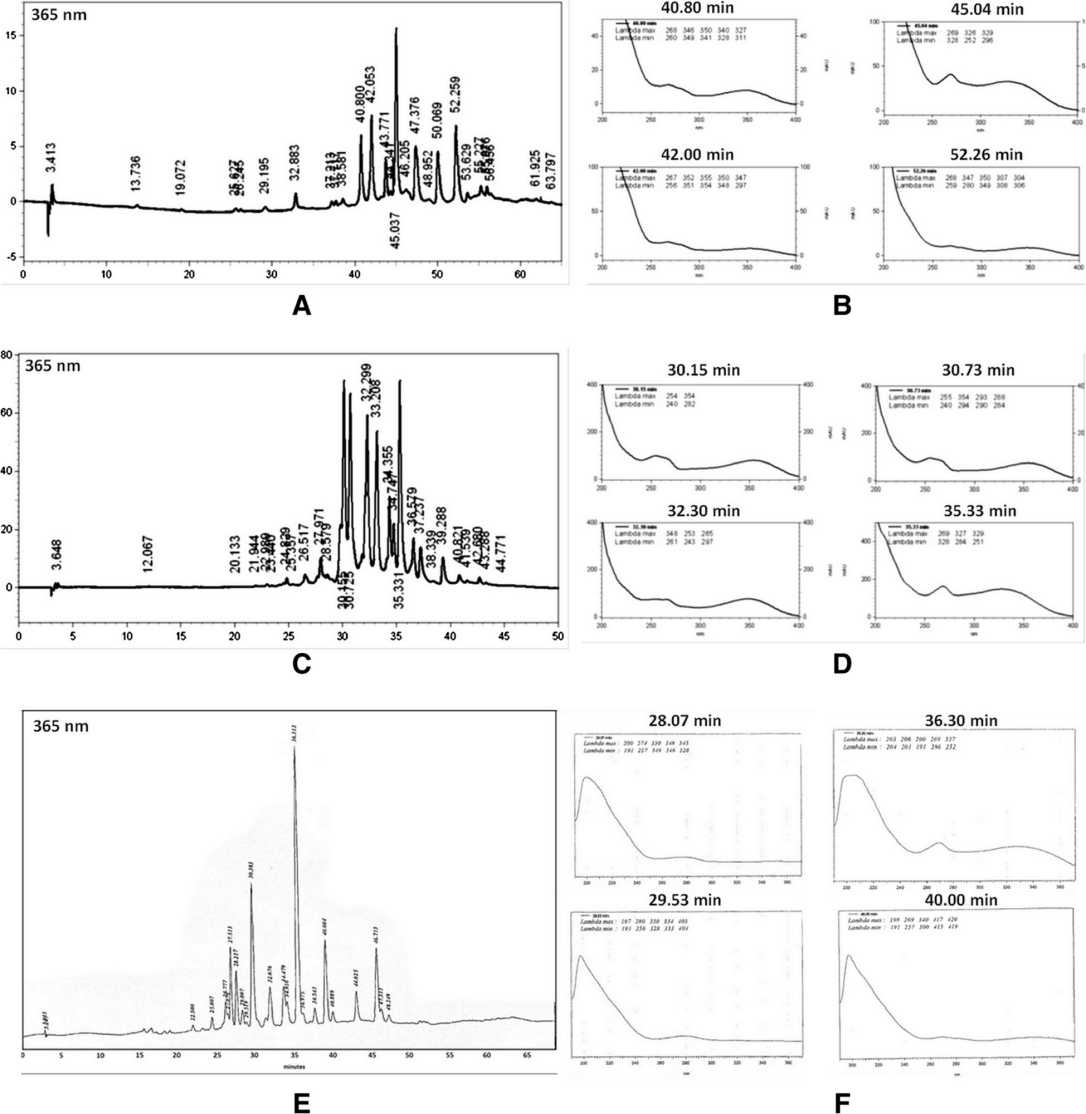
**HPLC-DAD analysis detects flavonoids as major compounds in fractions from** ***C. australis*** **. (A)** EtOAc fraction from the leaves of *C. australis* showing flavonoids with RT >40 min. **(B)** UV spectra of flavonoids in the EtOAc fraction. **(C)** *n-*BuOH fraction from the leaves of *C. australis* showing flavonoids with RT <40 min. **(D)** UV spectra of flavonoids in the *n-*BuOH fraction. **(E)** EtOAc-Pp fraction from the leaves of *C. australis* showing flavonoids and tannins with 20 min < RT <50 min. **(F)** UV spectra of flavonoids and tannins in the EtOAc-Pp fraction.

HPLC-DAD-UV analysis of EtOAc, *n-*BuOH and EtOAc-Pp fractions indicated different flavonoid profiles. Flavonoid aglycones and flavonoid monoglycosides with retention time (RT) greater than 40 min., predominated in EtOAc fraction (Figure 1A and B), while flavonoid diglycosides, more polar compounds (30 min. < RT <40 min) (Figure 1C and D), predominated in *n-*BuOH fraction. HPLC-DAD analysis of EtOAc-Pp showed more polar compounds (20 min < RT <50 min), like tannins and flavonoid diglycosides (Figure 1E and F).

All the ESI(−)FT-ICR MS analyses (Table 1) were made in negative ion mode. The structures were suggested based on their ultra-high resolution and accuracy mass. Molecular formula (M) and double bond equivalent (DBE) were utilized to propose the presence of flavonols, flavones, and their glycosides and condensed tannins (dimer and trimer of flavan-3-ol). EtOAc fraction showed presence of flavonols (*m/z* 285, 301 and 315), condensed tannins (*m/z* 529, 545 and 561), flavonol glycosides (*m/z* 447 and 463) and flavone glycoside (*m/z* 477). All were detected in deprotonated form, [M – H]^−^ ion. From the *n-*BuOH fraction were proposed flavonol glycosides (*m/z* 447, 463, and 609) and flavone glycosides (*m/z* 477 and 593) while the EtOAc-Pp showed the presence of flavonols and flavanones (*m/z* 285, 289, 301 and 315), flavonol glycosides (*m/z* 447 and 463), flavone glycoside (*m/z* 477) and condensed tannins (*m/z* 513, 529, 545 and 769). The chemical structure of compounds identified is proposed in Table 1.

**Table 1:** **Proposed compounds from ESI(−)FT-ICR MS analyzes**

**Suggested compounds**	**Class of natural product**	**EtOAc fraction**	***n-BuOH*** **fraction**	**EtOAc-Pp**	***m/z***_***theoretical***_	***m/z***_***measured***_	**Molecular formula (M)**	**Error (ppm)**	**DBE**
Kaempferol	Flavonol	+	-	+	285.04046	285.04042	C_15_H_10_O_6_	0.41	11
Quercetin	Flavonol	+	-	+	301.03538	301.03538	C_15_H_10_O_7_	0.25	11
Rhamnetin/isorhamnetin	Flavonol	+		+	315.05103	315.05098	C_16_H_12_O_7_	0.33	11
Quercetin pentoside	Flavonol Monoglycoside	+	+	+	447.09329	447.10361	C_21_H_20_O_11_	0.38	12
Quercetin hexoside	Flavonol Monoglycoside	+	+	+	463.08820	463.08801	C_21_H_20_O_12_	0.38	12
Tricetin-4′-methoxy-3′-â-D-glucoside^1^	Flavone Monoglycoside	+	+	+	477.10385	477.10361	C_22_H_22_O_12_	−0.12	12
-	Flavan-3-ol dimer	+	-	+	529.15041	529.15024	C_30_H_26_O_9_	0.51	18
-	Flavan-3-ol dimer	+	-	+	545.14532	545.14519	C_30_H_26_O_10_		18
-	Flavan-3-ol dimer	+	-	-	561.14024	561.08601	C_30_H_26_O_11_	0.45	18
	Flavan-3-ol dimer	+	-	-	591.11441	591.09653	C_30_H_24_O_13_	0.40	19
Vicenin-2 kaempferol diglycoside^1^	Flavone diglycoside	-	+	+	593.15120	593.15104	C_27_H_30_O_15_	−0.08	13
Quercetin dihexoside	Flavonol diglycoside	-	+	+	609.14611	609.14591	C_27_H_30_O_16_	0.06	13
-	Flavan-3-ol trimer	-	-	+	769.22905	769.22868	C_45_H_38_O_12_	0.08	27
	Flavan-3-ol trimer	-	-	+	785.22397	785.22349	C_45_H_38_O_13_	0.08	27

^1^Purified compounds identified by NMR, DBE - double bond equivalent, + detected, − not detected, Mass error (ppm) = [(*m/z*_measured_ – *m/z*_theoretical_)/*m/z*_theoretical_]*10^6^.

### Cytotoxicity and antiviral activity

**Figure 2:**
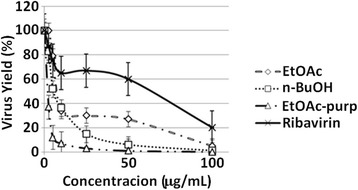
**Anti-MAYV activity of different fractions from** ***C. australis*** **.** The anti-MAYV activity of EtOAc, *n-BuOH* and EtOAc-Pp fractions from *C. australis* was evaluated by treating MAYV-infected cells with 0–100 μg/ml of these fractions for 24 h, and then staining for viral plaque counting. The graph shows the results from three independent experiments. Data are presented as mean% virus yield (compared to untreated controls) ± SD.

EtOAc, *n-*BuOH and EtOAc-Pp fractions inhibited MAYV replication in Vero cells. EtOAc and *n-*BuOH fractions inhibited MAYV production, respectively, by more than 70% and 85% at 25 μg/mL. EtOAc-Pp fraction inhibited MAYV production by more than 90% at 10 μg/mL. The antiviral ribavirin were much less potent inhibitors of MAYV replication, with IC_90_ values above 100 μg/mL (Table 2 and Figure 2).

**Table 2:** **Cytotoxicity and anti-MAYV activity of EtOAc,** ***n-BuOH*** **and EtOAc-Pp fractions**

**Substance**	**CC**_**50**_ ** (μg/mL)** ^**a**^	**CC**_**90**_ **(μg/mL)** ^**a**^	**IC**_**50**_ **(μg/mL)** ^**b**^	**IC**_**90**_ **(μg/mL)** ^**b**^	**SI** ^**c**^	**RP** ^**d**^
***n-BuOH***	2614 ± 366	821 ± 115	7,1 ± 1,0	40,9 ± 5,7	**20**	**10**
**EtOAc**	457,7 ± 9,5	176,1 ± 3,5	8,2 ± 0,2	89,1 ± 4,4	2	1
**EtOAc-Pp**	324,1 ± 6,5	154,7 ± 3,1	2,5 ± 0,1	4,7 ± 0,3	33	16,5
**ribavirin**	523,1 ± 42,5	215,4 ± 6,2	62,3 ± 4,4	112,4 ± 8,2	2	nd

^a^50% and 90% cytotoxic concentration.

^b^50% and 90% inhibitory concentration of viral replication.

^c^Selectivity Index = standard IC_90_/substance IC_90._

^d^Relative Potency = ratio between ribavirin (reference substance) IC_90_ and the tested substance’s IC_90_.

nd – Not determined.

The Selectivity Index (SI) and the Relative Potency are important indexes that can represent how suitable a substance is for further studies. EtOAc-Pp had the highest SI and 16 times higher Relative Potency than ribavirin. Although this is very relevant data, further studies need to be accomplished in order to address the use of these compounds as antivirals.

## Discussion

Since the three fractions tested have phenolic derivatives such as flavonoids and tannins as the major compounds, their antiviral activity can be attributed to the presence of them. EtOAc has flavonoid aglycones and flavonoid monoglycosides as major phenolics compounds, while in *n-*BuOH flavonoid diglycosides are the major ones. For the fraction EtOAc-Pp, beyond flavonoid mono and diglycosides, condensed tannins are present.

The presence of condensed tannins (flavan-3-ol, dimers and trimers) may be one of the factors responsible for antiviral activity. Tannins are known for their property of complexing with proteins, including lipo- and glycoproteins. Previous studies have reported that the binding of polymeric condensed tannins with protein was stronger than that of low molecular weight oligomers and monomers. It is believed that hydrogen bonding is an important factor in the binding of condensed tannins gelatin [34].

We know that the effect of the astringent polyphenols, including flavonoids and tannins, is dependent on the affinity of these substances with the protein and due to this is greatly influenced by the composition of each protein, as well as their hydrophilicity, therefore different viruses react polyphenols to different manner. In addition, previous works suggest the tanni*n-*like proanthocyanidins may link the protein covalently [35].

Takechi et al. [36] concluded in his work more highly condensed tannins have a greater antiviral activity, although the galloyl group contributes more to activity than the degree of condensation. It is known that the presence of o-dihydroxyphenyl group is related to the formation of protein-polyphenol complex. Moreover, it is thought that tannins interact with the protein particles from the surface of the host cell of the virus, as well as to the viral envelope [36].

In a previous study, Ferreira *et al*. found that the flavonoids quercetin group had a strong antiviral activity against MAYV, suggesting that this virus has proteins that are able to interact with phenolic substances from the group of flavonoid envelope [37].

Condensed tannins have been tested for their antiviral activity and exhibited antiviral activity against respiratory syncytial virus (RSV), influenza A virus (FLU-A) and parainfluenza virus (PIV). It also inhibited the growth of herpes viruses types 1 and 2 (HSV-1, HSV-2) and hepatitis A and B viruses. The proposed mechanism of action was from its connection with the viral envelope proteins, inhibiting the binding and penetration of the virus in the plasma membrane [38].

Yang *et al.* [39] compared several polyphenols derived from tea against influenza A and B and concluded that condensed tannins were the most active against the influenza A virus than monomeric polyphenols: theaflavin, procyanidin B-2 and procyanidin B-2. To evaluate the structure-activity relationship, they concluded that the dimers as theaflavin and procyanidin B-2, are more active against influenza A and B than the catechin monomers, such as (−)-EC and (±)-catechin and that galoyl group present in theaflavindigallate and procyanidin B-2 digallate not help on antiviral effect, probably due to the steric effect [39].

Since the trimers of tannins are only present in EtOAc-Pp, we correlate this to the greater antiviral activity of this fraction. Previous studies have shown that the degree of condensation is an important factor [36,39], being more highly condensed tannins more active; we believe that these substances are responsible for anti-viral activity.

## Conclusions

Our results show that *C. australis* is a valuable source of phenolics derivates with antiviral activity against the arbovirus MAYV. Although antiviral activity of tannins and other phenolics derivates are very common, this is the first report of anti-MAYV activity for these substances and this species. Our data are an important step in the evaluation of natural products as sources of novel drugs to be used in combination therapy, to circumvent drug resistance, or to replace currently used antivirals with unwanted cytotoxic effects.

## References

[1] Vasconcelos PFC, Rosa APAT, Pinheiro FP, Shope RE, Rosa JFST, Rodrigues SG, Dégallier N, Travassos da Rosa ES, Rosa APAT, Vasconcelos PFC, Rosa JFST (1998). Arboviruses pathogenic for man in Brazil. An overview of arbovirology in Brazil and neighbouring countries.

[2] Coimbra TLM, Santos CLS, Suzuki A, Petrella SMC, Bisordi I, Nagamori AH, Marti AT, Santos RN, Fialho DM, Lavigne S, Buzzar MR, Rocco IM (2007). Mayaro virus: imported cases of human infection in São Paulo state, Brazil. Rev Inst Med Trop Sao Paulo.

[3] Pinheiro FP, Travassos Da Rosa APA, Travassos Da Rosa JFS, Ishak R, Freitas RB, Gomes MLC, Le Duc JW, Oliva OFP (1981). Oropouche virus. I. A review of clinical, epidemiological, and ecological findings. Am J Trop Med Hyg.

[4] Pinheiro FP, Freitas RB, Rosa JFT, Gabbay YB, Mello WA, LeDuc JW (1981). An outbreak of Mayaro virus disease in Belterra, Brazil. I. Clinical and virological findings. Am J Trop Med Hyg.

[5] Vasconcelos PF, Travassos da Rosa AP, Rodrigues SG, Travassos da Rosa ES, Dégallier N, Travassos da Rosa JF (2001). Inadequate management of natural ecosystem in the Brazilian Amazon region results in the emergence and reemergence of arboviruses. Cad Saude Publica.

[6] Receveur MC, Grandadam M, Pistone T, Malvy D (2010). Infection with Mayaro virus in a French traveller returning from the Amazon region, Brazil. Euro Surveill.

[7] Brasil, Ministério da Saúde. **Ministério da Saúde intensifica medidas de controle da febre Chikungunya**. Accessed 11.03.2014. [http://portalsaude.saude.gov.br/index.php/o-ministerio/principal/secretarias/svs/noticias-svs/14667-ministerio-da-saude-intensifica-medidas-de-controle-da-febre-chikungunya]

[8] Brasil, Ministério da Saúde. **Saúde atualiza situação do vírus Chikungunya**. Accessed 11.03.2014. [http://u.saude.gov.br/d0ya9751]

[9] George DR, Finn RD, Graham KM, Sparagano OAE (2014). Present and future potential of plant-derived products to control arthropods of veterinary and medical significance. Parasit Vectors.

[10] Yasuhara-Bell J, Yuanan L (2010). Marine compounds and their antiviral activities. Antiviral Res.

[11] Newman DJ, Cragg GM (2012). Natural products as sources of new drugs over the 30 years from 1981 to 2010. J Nat Prod.

[12] Irwin HS, Barneby RC (1982). The American Cassiinae: a synoptical revision of Leguminosae tribe Cassieae subtribe Cassiinae in the New World. Mem New York Bot Gard.

[13] Wiersema JH (1989). A new name for a Brazilian Senna (Leguminosae: Caesalpinoideae). Taxon.

[14] Viegas C, Rezende A, Silva DHS, Castro-Gambôa I, Bolzani VS, Barreiro EJ, Miranda ALP, Moreira MSA, Young MCM (2006). Aspectos químicos, biológicos e etnofarmacológicos do gênero *Cassia*. Quim Nova.

[15] Nsonde Ntandou GF, Banzouzi JT, Mbatchi B, Elion-Itou RDG, Etou-Ossib AW, Ramos S, Benoit-Vical F, Abena AA, Ouamba JM (2010). Analgesic and anti-inflammatory effects of *Cassia siamea* Lam. Stem bark extracts. J Ethnopharmacol.

[16] Guzmán E, Pérez C, Zavala MA, Acosta-Viana KY, Pérez S (2008). Antiprotozoal activity of (8-hydroxymethylen)-trieicosanyl acetate isolated from *Senna villosa*. Phytomedicine.

[17] Lombardo M, Kiyota S, Kaneko TM (2009). Aspectos étnicos, biológicos e químicos de *Senna occidentalis* (Fabaceae). Rev Ciênc Farm Básica Apl.

[18] Longuefosse JL, Nossin EJ (1996). Medical ethnobotany survey in Martinique. J Ethnopharmacol.

[19] Franco EAP, Barros RFM (2006). Uso e diversidade de plantas medicinais no Quilombo Olho D’água dos Pires, Esperantina, Piauí. Rev Bras Plantas Med.

[20] Jones L, Bartholomew B, Latif Z, Sarker SD, Nash RJ (2000). Constituents of *Cassia laevigata*. Fitoterapia.

[21] Silva ALG, Ormond WT, Pinheiro MCB (2002). Biologia floral e reprodutiva de *Senna australis* (Vell.) Irwin & Barneby (Fabaceae, Caesalpinioideae). Bol Mus Nac NS Bot.

[22] dos Santos RN, Silva MGV, Braz FR (2008). Constituintes químicos do caule de *Senna reticulata* Willd. (Leguminoseae). Quim Nova.

[23] Barbosa FG, Oliveira MCF, Braz-Filho R, Silveira ER (2004). Anthraquinones and naphthopyrones from *Senna rugosa*. Biochem Syst Ecol.

[24] Hennebelle T, Weniger B, Joseph H, Sahpaz S, Bailleul F (2009). *Senna alata*. Fitoterapia.

[25] Kanno M, Shibano T, Takido M, Kitanaka S (1999). Antiallergic agent from natural sources. 2. Structures and leukotriene release-inhibitory effect of torososide B and torosachrysone 8-O-6″-malonyl beta-gentiobioside from *Cassia torosa* Cav. Chem Pharm Bull.

[26] Dehmlow EV, van Ree T, Guntenhöner M (1998). 2,4-trans-,7 4′-dihydroxy-4-methoxyflavan from *Cassia abbreviata*. Phytochemistry.

[27] Coetzeea J, Mcitekaa L, Malana E, Ferreira D (2000). Structure and synthesis of the first procassinidin dimers based on epicatechin, and gallo- and epigallo-catechin. Phytochemistry.

[28] Hatano T, Mizuta S, Ito H, Yoshida T (1999). C-Glycosidic flavonoids from *Cassia occidentalis*. Phytochemistry.

[29] Hatano T, Yamashita A, Hashimoto T, Ito H, Kubo N, Yoshiyama M, Shimura S, Itoh Y, Okuda T, Yoshida T (1997). Flavan dimers with lipase inhibitory activity from *Cassia nomame*. Phytochemistry.

[30] Viegas C, Bolzani VS, Furlan M, Barreiro EJ, Young MCM, Tomazela D, Eberlin MN (2004). Further bioactive piperidine alkaloids from the flowers and green fruits of *Cassia spectabilis*. J Nat Prod.

[31] Yuping T, Weiping Z, Fengchang L, Yanfang L, Jinghua W (2009). Flavone Glycosides from the Leaves of *Ginkgo biloba*. J Chin Pharmaceut Sci.

[32] Xie C, Veitch NC, Houghton PJ, Simmonds MSJ (2003). Flavone *C*-Glycosides from *Viola yedoensis* Makino. Chem Pharm Bull.

[33] Borenfreund E, Puerner JA (1985). Toxicity determined *in vitro* by morphological alterations and neutral red absorption. Toxicol Lett.

[34] Frazier RA, Deaville ER, Green RJ, Stringano E, Willoughby I, Plant J, Mueller-Harvey I (2010). Interactions of tea tannins and condensed tannins with proteins. J Pharm Biomed Anal.

[35] Gescher K, Kühn J, Lorentzen E, Hafezi W, Derksen A, Deters A, Hensel A (2011). Proanthocyanidi*n-*enriched extract from *Myrothamnus flabellifolia* Welw. exerts antiviral activity against herpes simplex virus type 1 by inhibition of viral adsorption and penetration. J Ethnopharmacol.

[36] Takechi M, Tanaka Y, Takehara M, Nonaka G, Nishioka I (1985). Structure and antiherpetic activity among the Tannins. Phytochemistry.

[37] dos Santos AE, Kuster RM, Yamamoto KA, Salles TS, Campos R, de Meneses MDF, Soares MR, Ferreira D (2014). Quercetin and quercetin 3-Oglycosides from *Bauhinia longifolia* (Bong.) Steud. show anti-Mayaro virus activity. Parasit Vectors.

[38] De Bruyne T, Pieters L, Deelstra H, Vlietinck A (1999). Condensed vegetable tannins: Biodiversity in structure and biological activities. Biochem Sys Ecol.

[39] Yang ZF, Bai LP, Huang WB, Li XZ, Zhao SS, Zhong NS, Jiang ZH (2014). Comparison of *in vitro* antiviral activity of tea polyphenols against influenza A and B viruses and structure–activity relationship analysis. Fitoterapia.

